# Extracellular Vesicles in Cancer Immune Microenvironment and Cancer Immunotherapy

**DOI:** 10.1002/advs.201901779

**Published:** 2019-10-23

**Authors:** Feng Xie, Xiaoxue Zhou, Meiyu Fang, Heyu Li, Peng Su, Yifei Tu, Long Zhang, Fangfang Zhou

**Affiliations:** ^1^ Institute of Biology and Medical Science Soochow University Suzhou 215123 P. R. China; ^2^ MOE Laboratory of Biosystems Homeostasis & Protection and Innovation Center for Cell Signaling Network Life Sciences Institute Zhejiang University Hangzhou 310058 P. R. China; ^3^ Key Laboratory of Head & Neck Cancer Translational Research of Zhejiang Province Zhejiang Cancer Hospital Hangzhou 310058 P. R. China

**Keywords:** anti‐tumor immunity, cancer immunotherapy, exosomes, extracellular vesicles, tumor microenvironment

## Abstract

Extracellular vesicles (EVs) are secreted by almost all cells. They contain proteins, lipids, and nucleic acids which are delivered from the parent cells to the recipient cells. Thereby, they function as mediators of intercellular communication and molecular transfer. Recent evidences suggest that exosomes, a small subset of EVs, are involved in numerous physiological and pathological processes and play essential roles in remodeling the tumor immune microenvironment even before the occurrence and metastasis of cancer. Exosomes derived from tumor cells and host cells mediate their mutual regulation locally or remotely, thereby determining the responsiveness of cancer therapies. As such, tumor‐derived circulating exosomes are considered as noninvasive biomarkers for early detection and diagnosis of tumor. Exosome‐based therapies are also emerging as cutting‐edge and promising strategies that could be applied to suppress tumor progression or enhance anti‐tumor immunity. Herein, the current understanding of exosomes and their key roles in modulating immune responses, as well as their potential therapeutic applications are outlined. The limitations of current studies are also presented and directions for future research are described.

## Graphical abstract

Exosomes derived from tumor cells and host cells play crucial roles in remodeling the tumor immune microenvironment, thereby determining the responsiveness of cancer therapies. In addition to being used as delivery carriers, exosomes are considered as noninvasive biomarkers for early detection and diagnosis of tumors. Exosome‐based therapies are emerging as promising strategies to suppress tumor progression or enhance anti‐tumor immunity.

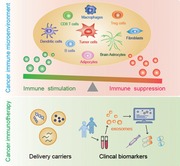

## 1 Introduction

The term exosome was first identified in 1981 as shedding vesicles with 5′‐nucleotidase activity derived from various normal and neoplastic cell lines.^1^ They belong to the family of extracellular vesicles (EVs), which include microvesicles (MVs, also known as microparticles and ectosomes), apoptotic bodies and exosomes.^2^, ^3^ Since early publications often did not discriminate between exosomes and MVs, we use the term “exosomes” only in those instances where a precise definition of exosomes is provided by the quoted references. Exosomes mainly differ from microvesicles in their size and mechanism of generation. Exosomes (30–150 nm in diameter) are released by exocytosis, whereas microvesicles (100–1000 nm in diameter) are secreted by shedding or outward budding of the plasma membrane. Apoptotic bodies (50–5000 nm in diameter) are released by dying cells during the later stages of apoptosis. A detailed morphological analysis has been performed on exosomes from single cell type. According to their size and shape, exosomes can be classified into nine different subpopulations or categories, implying that exosomes derived even from a single cell line are also morphologically diverse and probably functionally different.^4^ By employing asymmetric flow field‐flow fractionation (AF4), two exosome subpopulations (large exosome vesicles, Exo‐L, 90–120 nm; small exosome vesicles, Exo‐S, 60–80 nm) and an abundant population of non‐membranous nanoparticles termed “exomeres” (≈35 nm) were identified recently.^5^ Their findings indicate that all three nanosized particles have distinct size and cargo heterogeneity with diverse protein, lipid, RNA, and DNA profile. According to current research results, EVs are heterogeneous and the EV nomenclature is not yet established. The discriminations of EV subsets are still pretty much arbitrary. Although annexin A1 was proposed to be a specific marker for microvesicles that are shed directly from the plasma membrane,^6^ the unanswered question still remains, that is, what could be the specific markers for distinguishing exosomes from other types of microvesicles? Related to these issues, we would also like to question the composition characteristics and markers for different subpopulations of exosomes, such as exomere.^7^ It has been well shown that almost all living cells can secrete exosomes and they have been found in a number of human body fluids such as the blood plasma, urine, saliva, and breast milk.^8^, ^9^, ^10^, ^11^ The density of exosome is between 1.13 and 1.19 g mL^−1^,^12^, ^13^ and the size distribution and concentration can be measured by nanoparticle tracking analysis (NTA).^14^ In addition, exosomes can be visualized with a cup‐like morphology and have spherical structures consisting of a lipid bilayer by scanning electron microscopy (EM).^15^, ^16^, ^17^ Exosomes are considered as miniature versions of a parent cell not only because they have the same lipid bilayer membrane as the donor cell and carry a rich cargo of proteins, RNA, lipids, and DNA from donor cells, but also because their function are closely related to and reflecting the characteristics of parent cells.^3^, ^18^, ^19^, ^20^, ^21^, ^22^

As a mediator of intercellular communication, EVs play vital roles in many aspects of cellular homeostasis, physiology, and pathobiology.^23^, ^24^, ^25^, ^26^ In the context with tumor, EVs derived from cancer cells, immune cells, and also other non‐immune host cells serve as critical component of the tumor microenvironment (TME). EVs that secreted from different origins play distinct roles in tumor immunity, resulting in either increased or decreased tumor proliferation, metastasis, and drug resistance.^3^ Investigations in the past decades have led to the mysterious iceberg of exosomes beginning to fuse and reveal their important functions. As instance, the exosomes mediated transfer of tumor‐specific/enriched major histocompatibility complex (MHC) molecules and antigens that contribute to the antigen presentation, thereby facilitating immune recognition.^27^ However, the continuous release of exosomes from tumors can cause severe immunosuppression and inflammatory, which also endows tumor‐derived exosomes (TEX) with the capacity to predict tumor progression and prognosis.^26^ Tumor associated host cells, such as fibroblasts, adipocytes, and astrocytes tend to support metastasis by also releasing EVs,^28^, ^29^ while the EVs derived from immune cells, such as B cells, dendritic cells (DCs), and macrophages, mainly promote anti‐tumor immune responses.^12^, ^30^, ^31^ Therefore, a deeper and more comprehensive understanding on how exosomes could be integrated between tumor cells and the host microenvironment, as well as on how exosomes could be employed to develop novel therapeutic strategies, is of great importance for cancer treatment. In this review, we provide basic knowledge about exosomes and focus on the latest advances in exosome‐involved interplays between tumor cells, stroma cells, and the host anti‐tumor immunity.

## 2 Molecular Composition of Exosomes

**Figure 1:**
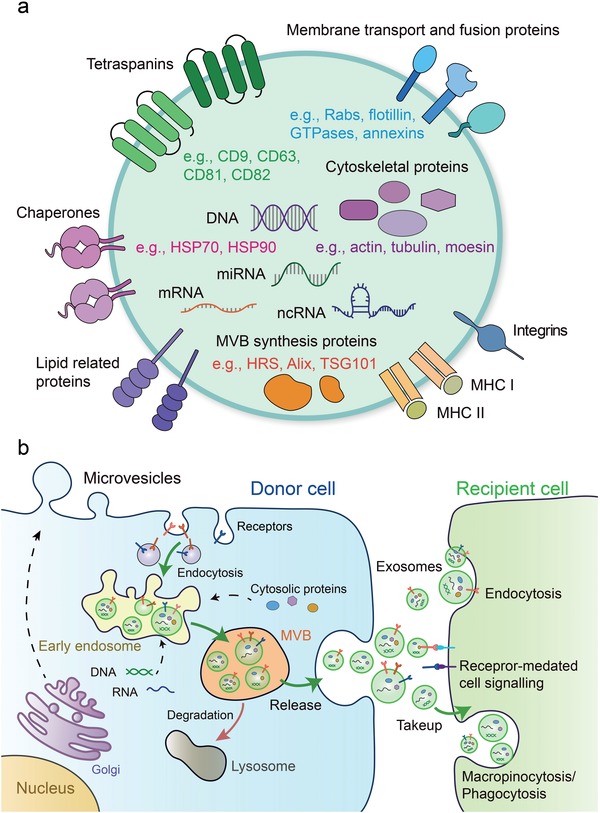
Molecular composition, biogenesis, secretion, and uptake of the exosomes. a) Exosomes contain complex contents including proteins, mRNA, miRNA, ncRNA, and DNA. TSG101 and Alix are involved in the formation of internal vesicles of MVBs. The tetraspanins such as CD9, CD63, and CD81, are the markers currently used to characterize exosomes. b) Exosomes originate from ILVs in MVBs. Firstly, proteins are transported from the Golgi or internalized from the cell surface, and nucleic acids should be endocytosed and transferred into the early endosomes. Then early endosomes maturate into late endosomes/MVB, which follow either the secretory or the degradative pathway. Microvesicles are released after formation by budding from the cytomembrane. Once released, exosomes can interact with recipient cells by direct signaling through ligand/receptor molecules on their respective surfaces. Exosomes can also be taken up by recipient cells via different manners such as direct fusion of their membrane, endocytosis, macropinocytosis, and even phagocytosis (right). Thus, exosomes function as a mode of intercellular communication and molecular transfer.

According to the exosome database (http://www.exocarta.org), 9769 proteins, 3408 mRNAs, 2838 miRNAs, and 1116 lipids are listed in the latest update.^32^ Exosome protein composition varies depending on the origin cell or tissue types.^33^ The proteins found in exosomes include membrane transport and fusion proteins (e.g., GTPases, annexins, flotillin, Rab2, Rab7, Rab11), tetraspanins (e.g., CD9, CD63, CD81, CD82), chaperones (e.g., heat‐shock protein (HSP) 70, HSP90), adhesion (e.g., integrins), MHC (class I and II molecules), cytoskeletal proteins (e.g., actin, tubulin, moesin), multi‐vesicular body synthesis proteins (e.g., HRS, Alix, tumor susceptibility gene 101 (TSG101)) and lipid related proteins^2^, ^3^, ^33^, ^34^, ^35^, ^36^, ^37^ (**Figure** **1**a). According to their known functions, TSG101, Alix, HSP70, CD9, CD63, and CD81, specific proteins highly enriched in exosomes, are frequently used as markers to identify exosomes. Tetraspanins, composed of four transmembrane domains, were firstly identified in B cell‐derived extracellular vesicles.^38^ Lipid rafts, such as glycosylphos phatidylinositol anchored proteins (LBPA) and flotillin, are highly enriched in exosomes. In addition, metabolic enzymes, such as GAPDH, enolase 1, PKM2, and PGK1, and molecules involved in signal transduction, such as protein kinases, 14‐3‐3, and G proteins, have been detected (**Table** **1**; most of the listed proteins are also present in MVs).^6^, ^39^ Apart from proteins, exosomes also contain RNA, including mRNAs, microRNAs (miRNAs), non‐coding RNAs, and mitochondrial DNA^40^, ^41^ (Figure 1a).Actually, the mRNA and miRNA are the first classes of nucleic acids identified in exosomes.^40^, ^42^, ^43^, ^44^ Interestingly, small fragments of single‐stranded DNA and large fragments of genomic, double‐stranded DNA encompassing all chromosomes were also reported in exosomes.^45^, ^46^ However, it is worth noting that a recent study overturned the conclusion that small extracellular vesicles contain DNA in which the active secretion of cytosolic DNA has been shown to occur through an amphisome‐dependent but exosome‐independent mechanism.^6^ This research indicated that dsDNA in the extracellular environment is not associated with exosomes or any other type of small EVs, but is present intracellularly in CD63‐positive compartments of a size consistent with MVEs. In this way, it proposes a model of autophagy‐ and MVE‐dependent, but exosome‐independent, active secretion of dsDNA. The debate on this issue is likely due to the fact that early studies often did not discriminate between MVs and exosomes. So there is much confusion on the presence of DNA in EVs and it will be of utmost importance to correctly identify the compartment and secretion mechanisms of EVs. Unexpectedly, exosomes could also contain intact metabolites, including amino acids, lipids, and TCA‐cycle intermediates.^47^ After exosomes bind to target cells via ligands or adhesion molecules, a proportion remains on the cell membrane surface, whereas the rest are internalized by macropinocytosis or phagocytosis.^48^, ^49^, ^50^, ^51^, ^52^, ^53^, ^54^ Thus, exosomes are proposed as a novel mode of intercellular communication between different cell types.

**Table 1:** Protein families present in exosomes

Functional characteristics	Protein names (e.g.)
MVB synthesis	HRS^a)^, Alix, TSG101^a)^, Clathrin^a)^
Membrane transport and fusion	Rabs^a)^, Annexins^a)^, GTPases^a)^, Flotillins^a)^
Tetraspanins	CD9^a)^, CD63^a)^, CD81^a)^, CD82^a)^
Heat shock proteins	Hsp70, Hsp90^a)^, HSPA5^a)^, Cyclophilin A^a)^
Signal transduction	Protein kinases, 14‐3‐3^a)^, G proteins^a)^
Metabolic Enzymes	GAPDH^a)^, Enolase 1^a)^, PKM2^a)^, PGK1^a)^
Cytoskeletal proteins	Actin, Tubulin^a)^, Moesin^a)^, Cofilin 1^a)^, Myosin^a)^
Antigen presentation	MHC I^a)^, MHC II^a)^, CD86^a)^
Adhesion	Integrins^a)^, MFGE8^a)^
Lipid related proteins	Flotinllinl^a)^, LBPA

^a)^ Labeling proteins are also present in MVs.

## 3 Biogenesis, Secretion, and the Uptake of Exosomes

Exosomes are generated by inward budding of the endosomal membrane, resulting in the accumulation of intraluminal vesicles (ILVs) within large multivesicular bodies (MVBs).^20^, ^39^, ^55^, ^56^ In contrast, microvesicles are directly generated through the outward shedding or budding vesicles of the plasma membrane.^57^ Therefore, exosomes are basically derived from the endocytic pathway of donor cells: the transmembrane proteins such as internalized receptors or proteins that are transported from the Golgi, such as MHC cl ass II molecules, should be endocytosed first then transferred into the early endosomes, which maturate and differentiate into late endosomes/MVBs. Once the MVBs fuse with the plasma membrane, exosome release into the extracellular environment by exocytosis^20^, ^58^, ^59^, ^60^, ^61^ (Figure 1b). The endosomal sorting complex required for transport (ESCRT) is the most well‐established driver of early endosomes maturation and MVB formation.^62^, ^63^, ^64^, ^65^ The ESCRT machinery consists of the ESRT‐0, ‐I, ‐II and ‐III complexes and sorts ubiquitinated intracellular cargos, which are destined for lysosomal degradation, into MVBs.^66^, ^67^, ^68^, ^69^ It has been demonstrated that sorting cargo into multivesicular endosomes (MVEs) did not depend on the function of the ESCRT machinery, but required the sphingolipid ceramide. As a result, the release of exosomes was reduced after the inhibition of neutral sphingomyelinases.^70^ In addition to this, the mechanisms of exosome secretion have been extensively studied and the Ras‐related proteins in brain (Rab) family, including Rab11, Rab27A, and Rab27B, are well accepted as key regulators in exosome secretory pathway.^71^, ^72^, ^73^, ^74^ Rab27A has been shown to be involved in the fusion of the MVB to the plasma membrane and the size of MVEs was strongly increased upon Rab27a depletion,^75^ whereas MVEs were redistributed towards the perinuclear region by knocking down of Rab27b.^75^ It was recently discovered that deletion of Rab27A led to loss of exosomal PD‐L1 thus blocked tumor growth through stimulating anti‐tumor immunity.^76^

Moreover, it has been suggested that lysosomal function can regulate exosome biogenesis by altering the fate of MVBs.^77^ The reduction of NAD^+^‐dependent deacetylase Sirtuin 1 (SIRT1) expression was shown to decrease the protein level of the lysosomal vacuolar‐type H+ ATPase proton pump (ATP6V1A), resulting in a reduction of MVBs targeted for lysosomal degradation and the enlargement of MVBs fused with the plasma membrane to release exosomes.^78^ And the pseudokinase mixed lineage kinase domain‐like (MLKL), which triggers necroptosis upon its phosphorylation by the protein kinase RIPK3, has been show to contributes to endosomal trafficking and generation of EVs.^79^ This study also shows that the release of EVs containing RIPK3‐phosporylated MLKL antagonizes its necroptotic function, serving as a mechanism of self‐restraint.^79^ During exosome secretion, pyruvate kinase type M2 (PKM2) has been reported to promote exosome release from tumor cells by phosphorylating synaptosome‐associated protein 23 (SNAP‐23), which enables the formation of the soluble N‐ethylmaleimide‐sensitive fusion factor attachment protein receptor (SNARE) complex to allow exosome release.^80^, ^81^, ^82^ Exosome secretion can also be modulated by cell interactions. In the process of rapid and efficient antigen presentation and immune activation, peptide‐MHC class II complexes (pMHC‐II) on the B cells associates with T cell antigen receptor (TCR) on antigen‐specific T cells, which in turn allow the CD4 T cells to recognize and activate B cells, meanwhile stimulating pMHC‐II to escape intracellular degradation and increase the secretion of pMHC‐II into B cell exosomes, leading to constant stimulation of T cells.^83^, ^84^

Exosomes that released into TME and body fluids could be taken up by recipient cells. Therefore, various biomolecules derived from exosomes can functionally regulate multiple cellular processes in their target cells.^40^ Exosomes can interact with their recipient cells by direct signaling via the interaction of ligand and receptor molecules on their respective surfaces. They can also be taken up by recipient cells through direct fusion of their membrane in different manners such as lipid raft‐, calveolae‐, and clathrin‐dependent endocytosis, macropinocytosis and phagocytosis.^52^, ^53^, ^54^, ^85^, ^86^, ^87^ In many cases, exosomes are fused with membrane and internalized together with phagocytic tracers. Dynamin2 (Dyn2), a key regulator of phagocytosis, is essential for exosome uptake.^54^ However, other reports suggest that exosomes are mainly internalized through non‐clathrin dependent, lipid raft‐mediated endocytosis rather than membrane fusion.^86^ The uptake of exosomes is negatively regulated by the lipid raft‐associated protein caveolin‐1 (CAV1) that depends on the ERK1/2‐HSP27 signaling.^86^ The transmission of exosomes in the body is known to have tissue‐ and organ‐specificity. Different integrins that expressed on TEXs is proved to dictate exosome adhesion to specific cell types and extracellular matrix molecules in particular organs.^88^ However, it is still under intense investigation and remains largely unclear what are the components in the exosomes that determine their organ‐specific location or cell type specificity. Likewise, much less is known about the difference between exosomes and MVs uptake. Collectively, the knowledge of vesicles biogenesis, secretion and uptake is not complete and deserve further exploration.

## 4 Exosome Purification and Characterization

In order to provide insights into the physiological function of exosomes, the purification and quantification of exosomes is critical to meet the demands of basic research and clinical applications. There are mainly five groups of exosome isolation techniques: ultracentrifugation (UC), size‐dependent methods such as ultrafiltration and size exclusion chromatography (SEC), density‐based separation, immune‐affinity capture methods and precipitation (**Table** **2**).^89^, ^90^, ^91^, ^92^ Each technique bases on one particular trait of exosomes, such as their morphology, density, size, or surface proteins. A list of advantages and disadvantages of each exosome isolation technique is summarized in Table 2. Among them, UC is the most traditional and widely accepted technology. In the UC process, a low‐speed centrifugation step (500 × *g* for 10 min) is firstly used to discard floating cells, and a subsequent higher‐speed centrifugation step (2000 × *g* for 20 min) is applied to remove the dead cells. Then, a higher‐speed centrifugation step (10 000 × *g* for 30 min) is needed to eliminate larger microvesicles and debris. A final ultracentrifugation (120 000 × *g* for 70 min, twice) allows collection of the precipitated exosomes.^91^, ^93^ For more details, we refer the reader to our recent review (https://doi.org/10.1002/smtd.201800021).

**Table 2:** Exosome isolation methods

Isolation methods	Purity	Principle	Major advantages	Major disadvantages
Ultracentrifugation	High	Density and size‐based	Large sample capacity	Cost time, high speed may damage exosomes
Density‐gradient centrifugation	High	Density‐based	High purity	Cost time, multi‐step procedures
Ultrafiltration	Moderate	Size‐based	Easy and fast	Filter membrane induced exosomes loss
Immune‐affinity capture	High	Specific markers on exosome	High specificity	High reagent cost, low efficiency
Precipitation	Low	Solubility or dispersibility	High efficiency	Containing non‐exosomal contaminants

Exosomes are commonly purified from cell culture supernatants or blood plasma and identified by physical and morphological characteristics.^8^, ^94^, ^95^ Typically, western blot, flow cytometry (FACS), and mass spectra analysis identify complex proteins in exosomes from different sources.^96^ Moreover, exosomes can be characterized by NTA,^97^ resistive pulse sensing (RPS), FACS, and EM. Comparison of these characterization technologies, along with their advantages and disadvantages, are shown (**Table** **3**).

**Table 3:** Exosome characterization technologies

Characterization technologies	Size range	Principle	Major advantages	Major disadvantages
Nanoparticle tracking analysis (NTA)	10 nm–2 µm	Dynamic light scattering, Brownian motion	High accurate, fluorescent samples	Multiple steps in preparation
Resistive pulse sensing (RPS)	30 nm–1 µm	Impedance, pulse signal	Homogenous, no need to isolate exosomes	Pore blocking
Flow cytometry (FACS)	30 nm–1 µm	Fluorescence detection	Subpopulation of a certain type of exosome by different surface markers, fluorescent samples	Based on the aldehyde‐sulfate latex beads
Electron microscopy (EM)	0.1 nm–100 µm	Cryo‐electron microscopy	Direct visualization and observation of exosomes purified or without purified in cells	Strict sample preparation procedures, interference of impurities

## 5 Functions of TEXs in Immune Environment

**Figure 2:**
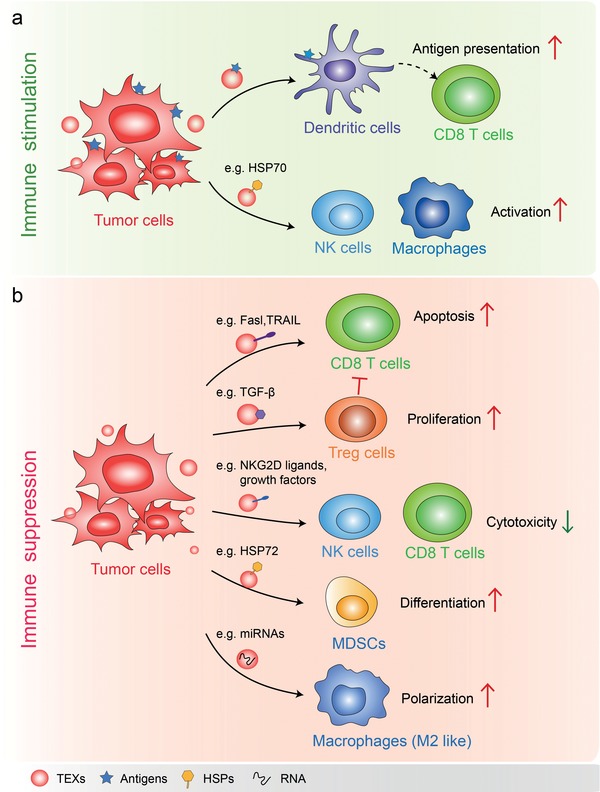
Functions of TEXs in tumor immune environment. a) TEXs present tumor antigen and enhance anti‐tumor immunity: in the presence of dendritic cells, TEXs loaded with specific antigens are capable of promoting the activation of tumor antigen‐specific CD8^+^ cytotoxic T‐lymphocytes. The HSP70 surface‐positive TEXs stimulate migratory and cytolytic activity of NK cells and macrophages. b) In most cases, TEXs function as immune suppressor. For instance, TEXs containing Fasl or TRAIL induce the apoptosis of T cells and suppress activation of T cells. TEXs bearing TGF‐β increase the proliferation of Treg cells which suppress immune responses. TEXs expressing NKG2D ligands or TGF‐β1 can inhibit the cytotoxicity of NK cells and CD8 T cells by triggering down‐regulation of their surface NKG2D expression. HSP72 bearing TEXs trigger STAT3 activation in MDSCs and promote MDSCs suppressive functions. TEXs containing miRNAs such as miR‐21‐3p, miR‐125b‐5p, miR‐181d‐5p, and miR‐1246 remodel macrophages to a tumor‐promoted phenotype.

In the TME, immune cells including T cells, B cells, macrophages and dendritic cells frequently infiltrate the tumor tissue and interact with tumor and stroma cells. Via secreting TEXs, tumor cells could deliver immune‐stimulatory or immune‐suppressive signaling molecules therefore regulate the development, maturation, and anti‐tumor capacity of targeted immune cells^3^, ^26^, ^98^, ^99^ (**Figure** **2**).

TEX can carry multiple tumor antigens, which are efficiently taken up and cross‐presented by MHC‐I molecules on dendritic cells in a human in vitro model system.^100^ It is likely that TEXs may contain specific receptors or ligands for efficient uptake by antigen presenting cells (APCs). However, the in vivo relevance of TEXs needs to be validated. These tumor antigen‐loaded DCs can increase the tumor antigen‐specific CD8^+^ cytotoxic T‐lymphocytes (CTLs), thus enhancing immune responses.^100^, ^101^ Of notable interest, the direct activation of T cells by cancer exosomes has not been reported, CD8^+^ cytotoxic T‐cell stimulatory function of cancer exosomes requires uptake and processing tumor antigens by DCs.^100^, ^102^, ^103^

In addition, TEXs that also bear HSP70, as well as other specific tumor antigens, promote the migratory and cytolytic activity of NK cells and TNF‐α production by macrophages^104^, ^105^ (Figure 2a). Bcl‐2‐associated athanogene 4 (Bag‐4), as an anti‐apoptotic protein, was found to interact with HSP70 not only in the cytosol but also on the plasma membrane.^106^ HSP70/Bag‐4‐positive exosomes from pancreas and colon carcinoma stimulate migration and cytolytic activity in NK cells which could be completely abrogated by HSP70‐specific antibody.^104^ Subsequent research indicated that HSP70 was released into the extracellular environment in a membrane associated form which more effectively induce TNF‐α production as an indicator of macrophage activation, as compared with free recombinant protein.^105^

However, for the most part, TEXs have been shown to promote immunosuppressive and pro‐tumorigenic effects^107^, ^108^, ^109^, ^110^, ^111^ (Figure 2b). In fact, in the TME, TEXs carrying native tumor antigens may not “efficiently” transfer these antigens to DCs for processing and cross‐presentation. More recent evidences support the conclusion that TEXs could assist cancer cells and reflect the aims and functions of the parent cancer cell: that is, to survive, grow and metastasize. For example, TEXs of melanomas were demonstrated to reprogram bone marrow progenitor cells toward a pro‐vasculogenic phenotype in the pre‐metastatic niche.^99^ TEXs derived from prostate cancer cells express fas ligand (Fasl also known as CD95L), a T cell killing molecule that induces the apoptosis of T cells, thus act as systemic antigen presenting death signals of CD8^+^ T cells.^112^, ^113^ Similarly, TEXs bearing TRAIL also induce apoptosis of activated anti‐tumor T cells.^114^ As a critical cytokine that mediates suppression of CD8^+^ T cells and the proliferation of Foxp3^+^ regulatory T cells,^115^ Transforming growth factor‐β (TGF‐β) was shown to be transmitted via breast cancer exosomes.^116^ Furthermore, TEXs expressing ligands for NKG2D could reduce the cytotoxicity of natural killer (NK) cell and CD8 T cells by down‐regulating their surface NKG2D expression.^117^, ^118^ In addition, TEXs can also inhibit dendritic cell maturation by repressing the differentiation of myeloid precursors into DCs and the generation of myeloid‐derived suppressor cells (MDSCs).^110^, ^111^ A similar study also showed that HSP72 bearing TEXs activate STAT3 in MDSCs in a TLR2/MyD88‐dependent manner and thus mediate T cell–dependent immunosuppressive functions of MDSCs.^119^ Likewise, exosomes derived from hypoxic epithelial ovarian cancer cells that enriched with miRNAs, such as miR‐21‐3p, miR‐125b‐5p, and miR‐181d‐5p, potently induce the polarization of M2 macrophages with a pro‐tumor phenotype.^120^ And the exosomes derived from colon cancer cell was shown to be enriched with miR‐1246 that can reprogram neighboring macrophages into a tumor supportive and anti‐inflammatory state.^121^ As discussed above, TEXs can cause immune suppression by promoting the differentiation of inhibitory immune cells, including Treg, MDSCs, and M2‐like TAMs.

## 6 Mechanisms of TEXs in Modulating Innate and Adaptive Immunity

Functions of exosomes are determined by their specific content, in other words, depending on the cargos that are specifically delivered. It is possible that different TEX subtypes which containing specific context under certain physiological conditions mediate immunostimulatory or immuninhibitory activity.

**Figure 3:**
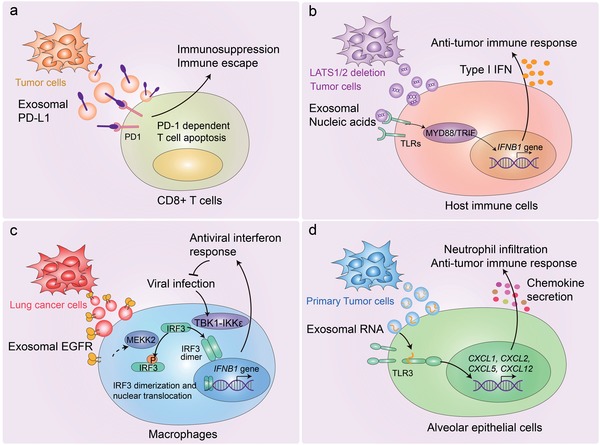
Mechanisms of TEXs in modulating innate and adaptive immunity. a) Tumor cell‐derived exosomal PD‐L1 can be transferred to CD8^+^ T cells, leading to the immunosuppression and immune escape in melanoma and prostate cancer. b) LATS1/2 deficient tumor cells secrete nucleic‐acid‐rich extracellular vesicles, which induces anti‐tumor immune responses via type I interferon. c) Tumor cell‐derived exosomal EGFR can be transferred into host macrophages to reduce their production of type I interferon and inhibit antiviral immunity. d) Primary tumor‐derived exosomal small nuclear RNAs can be transferred to the lung epithelial cells, leading to the activation of TLR3, production of chemokine, and recruitment of neutrophils. Thus, tumor‐derived exosomal small nuclear RNAs can elicit a pro‐metastatic inflammatory microenvironment by suppressing innate and adaptive anti‐tumor immunity.

Although the specific mechanisms by which tumor exosomes regulate host immunity are complicated and largely unknown, we summarized and discussed several recent important research findings to explain the enormous heterogeneity of immunomodulatory mechanisms in this section (**Figure** **3**). Programmed death‐ligand 1 (PD‐L1), a membrane bound ligand on many cancer cells, can bind programmed death‐1 (PD‐1) receptor on T cells to suppresses antigen‐derived activation of T cells and elicit the immune checkpoint response.^122^, ^123^ It has been found that PD‐L1 is also located on the surface of TEXs from plasma samples of patients with a variety of cancers.^124^ Two recent studies found exosomal PD‐L1 could play critical immunosuppressive roles in melanoma and prostate cancer^76^, ^125^ (Figure 3a). The circulating exosomal PD‐L1 suppresses the function of CD8^+^ T cells thus facilitate tumor growth. Remarkably, in patients with metastatic melanoma during anti‐PD‐1 therapy, the responders displayed a larger increase of exosomal PD‐L1 in comparison to the non‐responders. Therefore, the circulating exosomal PD‐L1 could be considered as a biomarker for the clinical outcomes of anti‐PD‐1 therapy.^125^ This was confirmed by another independent study which showed that exosomal PD‐L1 can suppresses T cell function and promotes tumor progression by inducing a systemic immunosuppression. Suppression of Exosomal PD‐L1 by depletion of Rab27A and nSMase2, two important exosomal biogenesis genes, induces systemic anti‐tumor immunity and memory.^76^ It is worth noting that in addition to PD‐L1, tumor exosomes should contain other proteins or RNAs that also exert immunosuppressive functions, awaiting further investigations. Moreover, by delivering different signals, TEXs can broadly affect the proliferation, apoptosis, cytokine production, and reprogramming of T cells.^126^ TEXs are able to induce apoptosis of activated CD8^+^ effecter T cells by activating the Fas/Fas ligand pathway and promote the expansion of Treg cells, thus contributing to immune suppression and tumor escape.^127^

Both the large tumor suppressor 1 (LATS1) and LATS2 are key kinases in Hippo pathway that controls organ development and mainly play tumor‐suppressive roles by targeting YAP/TAZ for proteasomal degradation.^128^, ^129^, ^130^ Unexpectedly, loss of LATS1/2 inhibits tumor growth and metastasis by enhancing immunogenicity of tumor cells and anti‐tumor immune responses. The nucleic‐acid‐rich extracellular vesicles (50–200 nm in diameter) secreted from LATS1/2 deficient tumor cells stimulate the host TLR‐MYD88/TRIF nucleic‐acid‐sensing pathways and promote the production of type I interferon, thus inducing adaptive immune responses.^131^ Further investigations are essential to explore the signaling mechanisms involved in unidentified proteins or nucleic acids in extracellular vesicles (Figure 3b). Several proteomic studies also identified Hippo pathway to be associated with endocytosis and vesicle trafficking,^132^, ^133^, ^134^ implying that Hippo pathway may regulate extracellular vesicles biogenesis.

Exosomes from normal human urinary are enriched for innate immune proteins thus function as immune effectors that contribute to host defense within the urinary tract.^135^ However, based on our research, exosomes derived from lung cancer cells mainly antagonize the host innate immunity compared with exosomes derived from normal lung fibroblasts.^136^ Our results shown that tumor exosome from lung cancer cells were able to transfer activated epidermal growth factor receptor (EGFR) to the host macrophages, in which the exosomal EGFR engaged with the macrophage‐intrinsic signaling pathway that reduced their production of type I interferons (IFNs) and antiviral immunity. In macrophages, the Serine/Threonine kinase MEKK2 serves as effecter that could be activated by TEX‐delivered EGFR. The stimulated MEKK2 directly phosphorylated Ser173 on IRF3, a transcription factor crucial for IFN‐β induction. This triggered K33‐linked poly‐ubiquitination of IRF3 on its nuclear‐localization sequence (NLS) thus blocked its dimerization, nuclear translocation, and transcriptional activity.^136^ This study explained the reason why tumors can interfere with the innate antiviral system via exosomes and identify a mechanism by which cancer cells can dampen host innate immunity (Figure 3c). In addition to this, TEXs contribute to metastasis through educating the pre‐metastatic niche. The small nuclear RNAs enriched in the TEXs, via activating Toll‐like receptor 3 (TLR3) in lung epithelial cells, could induce production of chemokines such as CXCL1, CXCL2, CXCL5, and CXCL12, therefore promote recruitment of neutrophil.^137^ Once recruited in the niche, neutrophils can switch to have tumor promoting roles thus drive metastasis^138^, ^139^ (Figure 3d). In line with this study, neutrophils were shown to elicit a pro‐metastatic inflammatory microenvironment by suppressing both innate and adaptive anti‐tumor immunity.^139^, ^140^, ^141^

We speculate that the TEXs derived from different cancer cells or the same cancer type but under different pathological conditions might be selective for recipient cells and function in specific molecular pathways. However, who or what determines which immune cell could be targeted by TEXs is an unresolved issue. Future studies are needed to elucidate the dual role of TEXs in cancer‐immune progression. Based on our preliminary observations, the components in TEXs are dynamically altered and closely related to the degree of malignancy of donor tumor cells. Thus it is likely that in the early stage of tumor, TEXs barely contain immune‐suppressive molecules but carry relevant tumor antigens to initiate immune responses via DCs. While in the late stage of tumor progression, malignant tumor cells could suppress the host's innate and adaptive immunity by releasing exosomes carrying abundant immunosuppressive factors such as PD‐L1 and EGFR.

## 7 Stroma Cells in the TME Support Tumor Progression via Secreting Exosomes

**Figure 4:**
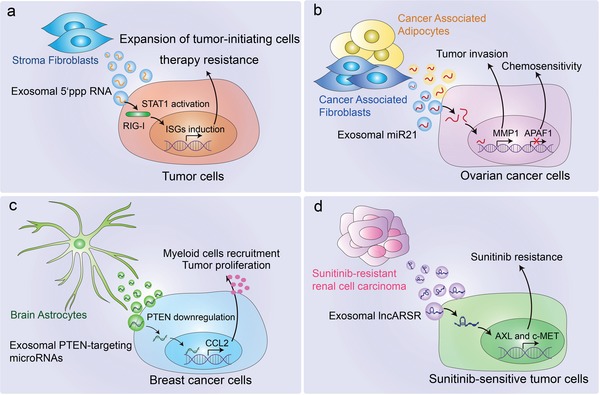
Stroma cells in the TME support tumor progression via secreting exosomes. a) NOTCH‐MYC signaling in stromal fibroblasts shed exosomes containing unshielded RN7SL1 RNA. Upon being transferred to breast cancer cells, unshielded RN7SL1 activates RIG‐I and STAT1, and increases ISG induction, resulting in tumor growth, metastasis, and therapy resistance. Upon being transferred to immune cells, it can also drive an inflammatory response by increasing the percentage of myeloid DC populations. b) Cancer‐associated adipocytes and fibroblast‐derived exosomal miR21 can be transferred to the cancer cells, which downregulate APAF1 expression and upregulate MMP1 expression, resulting in tumor invasion and chemoresistance. c) Brain astrocyte‐derived exosomal PTEN‐targeting microRNAs can be transferred to metastatic tumor cells, induce an increased secretion of the chemokine CCL2 and facilitate the recruitment of IBA1^+^ myeloid cells which promotes tumor outgrowth. d) Sunitinib resistant RCC cell‐derived exosomal lncARSR can be transferred to sensitive cells and facilitates AXL and c‐MET expression, thus disseminating sunitinib resistance.

The development of cancer metastases at distant organs requires disseminated tumor cells' adaptation to, and co‐evolution with the different microenvironments of the metastatic sites. Stromal cells in the tumor microenvironment regulate cancer progression, therapy resistance, inflammatory responses via interaction with cancer cells.^142^ Exosomes derived from stromal fibroblasts contains unshielded RN7SL1 RNA, which is 5′‐triphosphorylated. Upon transfer to breast cancer cells, unshielded RN7SL1 RNA activates the viral RNA pattern recognition receptor (PRR) RIG‐I, resulting in STAT1 activation and ISG induction. Then STAT1 amplifies the NOTCH3 transcriptional response, leading to tumor growth, metastasis, and therapy resistance. Upon transfer to immune cells, unshielded RN7SL1 drives an inflammatory response by increasing the percentage of myeloid DC populations which express maturation and activation markers^143^ (**Figure** **4**a). Advanced ovarian cancer frequently spreads to the visceral adipose tissue of the omentum. Exosomes derived from cancer‐associated adipocytes (CAAs) and fibroblasts (CAFs) contain high level of microRNA‐21 (miR21). By regulating apoptosis protease‐activating factor‐1 (APAF1) and MMP1 expression in the target ovarian cancer cells, exosomal miR21 derived from neighboring stromal cells can confer chemoresistance and an aggressive phenotype (Figure 4b). Except for nucleic acid, exosomes from CAFs also contain intact metabolites, including amino acids, lipids, and TCA‐cycle intermediates, which could be utilized by cancer cells for central carbon metabolism and tumor growth under nutrient deprivation or nutrient stressed conditions.^47^ Regardless of the types of tumor, brain metastasis has a particularly poor prognosis with high morbidity and mortality. Patients with brain tumor barely manage for survive more than a year and few effective treatment are currently available. Of note, tumor cells disseminate to the brain often show loss of PTEN, but not to other organs. Exploring results revealed that brain astrocyte‐derived exosomes mediate an intercellular transfer of PTEN‐targeting microRNAs to the metastatic tumor cells. Furthermore, such adaptive PTEN loss in brain metastatic tumor cells induces an increased secretion of the chemokine CCL2, facilitating the recruitment of IBA1^+^/CCR2^+^ myeloid cells at the micro‐metastasis site and enhancing the outgrowth of brain metastatic tumor cells^29^ (Figure 4c). It is well recognized that patients who develop resistance to drug have limited therapeutic options in the clinic. At present, sunitinib resistance appears to be the major challenge for advanced renal cell carcinoma (RCC). Recent studies have described the role of exosomes in the dissemination of drug resistance. Drug resistance is a major challenge for advanced RCC. It has been reported that highly abundant lncARSR (lncRNA activated in RCC with sunitinib resistance) is present in sunitinib‐resistant RCC cell‐derived exosomes. The exosomal lncARSR can be transferred to sensitive cells and facilitate AXL and c‐MET expression in RCC cells by competitively binding miR‐34/miR‐449, thereby disseminating sunitinib resistance^144^ (Figure 4d).

These results show that stromal or cancer‐associated normal cells reprogrammed in the TME utilize exosomal miRNAs or lncRNAs to induce resistance of tumor cells to drugs or chemotherapy. Based on this research and the fact that tumor exosomes are rich in RNA, we can be sure that there must be similar tumor resistance mechanisms transmitted by exosomal RNA, which needs to be resolved in the future. These identified exosomal RNAs are also therapeutic targets to overcome drug or chemotherapy resistance, enhancing the clinical benefits in patients. Thus, inhibition of specific RNA to block compensatory signaling pathways could resensitize resistant cancer cells to drug or chemotherapy and it is necessary to identify novel targets for resistance prevention and therapy. In addition, preventing the exosomal transfer of miRNA is another new strategy for conferring EV‐induced resistance to drugs or therapies. In summary, elucidating the molecular mechanism of EV‐induced resistance could contribute to the development of rationally designed combination cancer therapies.

## 8 Exosomes from Distinct Immune Cells Play Divergent Roles in Cancer Immunity

**Figure 5:**
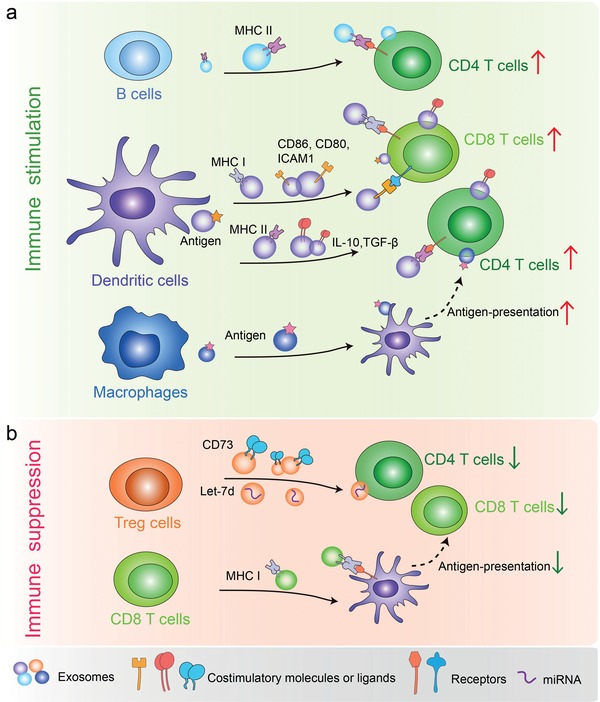
Exosomes from distinct immune cells play divergent roles in regulating cancer immunity. a) B cell‐derived exosomes bearing MHC II activate CD4^+^ T cells. DC‐derived exosomes containing tumor‐derived antigens, costimulatory molecules, and proteins, can promote the activation of CD4^+^ T cells and CD8^+^ T cells. Macrophage‐derived exosomes bearing MHC I can be transferred to DCs, thereby enabling them to activate antigen‐specific CD4^+^ T cells. b) Treg‐derived exosomes containing CD73 can inhibit T cell activation. CD8^+^ T cell‐derived exosomes carrying MHC I also mediate immune suppression by inhibiting the antigen presentation of DCs.

Exosomes derived from multiple types of immune cells broadly modulate antigen presentation and T cells function by playing immune‐stimulatory or immune‐suppressive roles, leading to highly efficient anti‐tumor immunity or tumor immune tolerance^30^, ^145^, ^146^, ^147^ (**Figure** **5**). It was first reported in 1996 that Epstein‐Barr virus‐transformed B cells secreted vesicles carrying MHC class II that could be presented to antigen‐specific T cells, inducing antigen‐specific MHC II‐restricted CD4 T cell responses^12^ (Figure 5a). In line with this, dendritic cell‐derived exosomes also contain MHC I/II and other tumor antigens that stimulate anti‐tumor immune response.^148^ Besides, exosomes from IL‐10‐treated DCs suppress inflammatory and autoimmune responses.^149^ Exosomes derived from TGF‐β1 gene‐modified bone marrow DCs have immunosuppressive function in inflammatory bowel disease by inducing regulatory T cells and decreasing the proportion of Th17 in lymphocytes.^150^ As outlined above, exosomes from APCs, such as B cells and DCs, contain specific peptides and antigens involved in activating antigen‐specific T cells.^151^, ^152^ However, free exosomes in vitro are not able to stimulate naive T cells, suggesting this process requires TCR crosslinking and T cell co‐stimulation.^153^, ^154^ Indeed, it increases the T cell stimulatory capacity after the interaction of exosomes with dendritic cells.^152^, ^153^ Moreover, DC‐derived exosomes also accumulate proteins such as CD80, CD86, and intercellular adhesion molecule 1 (ICAM1, also called CD45) which are involved in T cell co‐stimulation^48^, ^155^, ^156^, ^157^ (Figure 5a). Except for APCs, macrophage‐secreted exosomes could transfer their surface antigens to DCs in a ceramide‐dependent manner thereby promoting the activation of CD4^+^ T cells^31^ (Figure 5a). These findings revealed that exosomes function to mediate collaboration between macrophages and DCs for antigen presentation. In contrast, regulatory T (Treg) cells, a subset of CD4^+^ T cells, can suppress other T cells through cell‐contact dependent manner (cytolysis and inhibitory receptor engagement) or cell‐contact independent manner (IL‐2 consumption and suppressive cytokine secretions, such as TGF‐β and IL‐10).^158^ It is conceivable that exosomes derived from Treg cells also play immunosuppressive roles, similar with their donor cells.^159^ Exosomes from Treg cells contain CD25, CTLA‐4, and CD73. CD73‐positive Treg exosomes could convert extracellular denosine‐5‐monophosphate to adenosine. Once adenosine binds to its receptors on activated effector T cells, it leads to suppression of cytokine production and T‐cell responses.^159^ In addition to proteins, specific miRNA also contributes to the Treg exosome mediated suppression. For example, microRNA Let‐7d could be preferentially packaged into Treg exosomes and transferred to T helper 1 (Th1) cells, resulting in suppressed Th1 cell proliferation and IFN‐γ secretion.^160^ Th1 cells, a subtype of Naïve CD4 T cells, are capable of producing IFN‐γ which plays a central role in antitumor immunity.^161^ Furthermore, whether Treg cells package different RNA species or proteins in exosomes and deliver them to different Th cells such as T helper 2 (Th2) is yet to be clarified (Figure 5b). During cognate T cell‐DC interactions, several proteins, including MHC and co‐stimulatory molecules, are transferred from DCs to CD4 T cells, down‐regulating immune responses. One study has showed that exosomes derived from CD8^+^ T cells can be endocytosed by APCs through MHC‐I/TCR interactions and this inhibit DCs mediated antigen‐specific CD8^+^ CTL responses (Figure 5b).^162^ Overall, the current research on immune cell exosomes lags far behind the direction of tumor cell exosomes. To explore the composition, characteristics and functional proteins of different immune cell exosomes is of great significance for understanding the functions of immune cells, particularly may explain how immune cells can “communicate” over long distances.

## 9 Exosomes in Cancer Immunotherapy

Compared with other nano‐carriers, exosomes have high stability in circulation and intrinsic ability of horizontal cargo transfer and are less toxic and immunogenic. Due to the presence of CD47, a widely expressed integrin‐associated transmembrane protein on exosomes, they can effectively avoid phagocytosis by the circulating monocytes, thus promoting the delivery of their cargos. CD47 interacts with its ligand signal regulatory protein α (SIRPα, also known as CD172a) on macrophages, which induces a “don't eat me” signal that inhibits phagocytosis.^163^ On the other hand, unlike liposomes, exosomes contain the plasma membrane‐like phospholipids and membrane‐anchored proteins, which could contribute to their diminished clearance from the circulation. Due to their capacity to cross the blood‐brain barrier, exosomes are also employed to be a novel strategy in the treatment of brain tumor.^164^, ^165^ Therefore, the idea of using exosomes as a vehicle in clinical practice is promising and inspiring.^166^, ^167^ The modified exosomes are designed for clinical applications through artificially optimizing the integration of specific loadings such as tumor drugs and tumor targeting RNAi.^168^, ^169^

**Figure 6:**
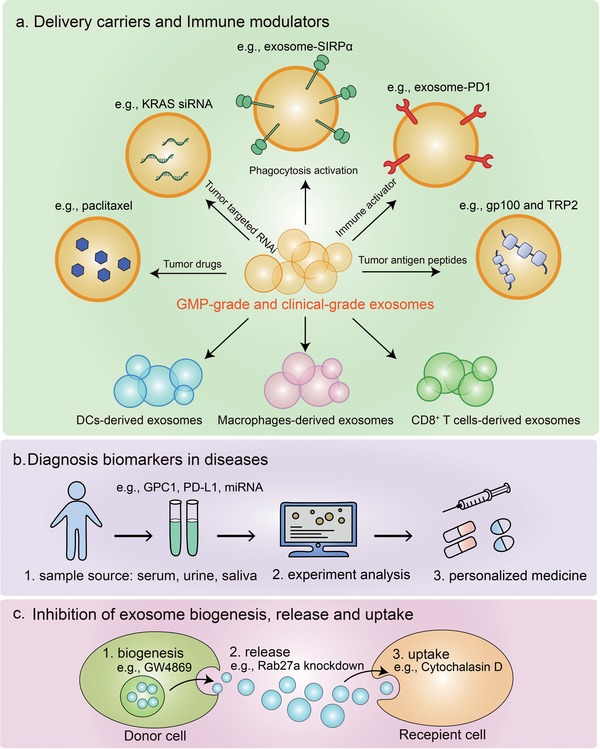
Exosomes in cancer immunotherapy. a) As exosomes have high stability in circulation and good capacity to transfer horizontal cargo, they have been explored as delivery carriers loaded with drugs or tumor targeted RNAi in different diseases. In addition, exosomes can be employed as immune modulators by expressing proteins such as SIRPα, PD1, or tumor antigen peptides. The exosomes derived from immune cells including DCs, macrophages and CD8^+^ T cells are demonstrated to stimulate anti‐tumor immune responses. Importantly, large‐scale generation of good manufacturing practice‐grade (GMP‐grade) and clinical‐grade exosomes are generated for clinical applications. b) Exosomes bearing GPC1, PD‐L1, or certain miRNA could be valuable as cancer biomarkers. c) Inhibition of exosomes biogenesis, release, and uptake is another strategy of cancer immunotherapy.

Paclitaxel is extensively applied as anti‐tumor drugs for various tumor types including breast cancer.^170^ Limited by the poor aqueous solubility of paclitaxel, it is an urgent need to develop new methods for increasing solubility and improving therapeutic efficacy. Solution was made by using exosomes as drug delivery carriers, in which paclitaxel‐packaging exosomes were observed to efficiently kill tumor cells.^171^ Similarly, exosomes loaded with chemotherapeutic drugs such as curcumin, methotrexate and cisplatin have promising anti‐tumor effects in treatment of a variety of cancers (**Figure** **6**a).^172^, ^173^, ^174^ In addition to delivering drugs, using exosomes as a siRNA delivery vehicle to silence oncogenes in tumor cells has been explored recently.^168^ The oncogenic activation of GTPase KRAS are commonly occurred in pancreatic cancer,^175^, ^176^ but the nucleic acids targeting KRAS have low stability and uncontrollable release in the blood circulation thus it remains a challenge to generate an effective therapy by targeting of KRAS. A reported, exosomes derived from normal human foreskin fibroblast can function as efficient carriers of KRAS siRNA, which significantly suppresses pancreatic tumors progression and enhances overall survival in mouse models^177^ (Figure 6a).

Owing to the lipid bilayer membrane, engineered exosomes can express transmembrane proteins on their surface and thus actively participate in tumor immunotherapy. By engaging SIRPα, CD47 limits the ability of macrophages to engulf tumor cells, which acts as a major phagocytic barrier.^163^ Based on this finding, exosomes designed to harbor SIRPα variants could function as immune checkpoint blockade that antagonizes the interaction between CD47 and SIRPα thus induce augmented tumor phagocytosis, leading to prime effective anti‐tumor T cell response^163^ (Figure 6a). In recent years, checkpoint blockade antibodies against PD‐1 or PD‐L1 have shown unprecedented clinical responses.^178^ Similar to the PD‐1/PD‐L1 blocking antibodies, cell membrane derived nanovesicles, as a bioengineering strategy, presenting PD‐1 receptors on their membranes could enhance anti‐tumor responses through disrupting the PD‐1/PD‐L1 immune inhibitory axis^179^ (Figure 6a). Moreover, exosome‐based tumor antigen‐adjuvant co‐delivery system for cancer immunotherapy is another engineering strategy to improve immunogenicity. For instance, engineered melanoma tumor cell‐derived exosomes containing endogenous tumor antigens (gp100 and TRP2) and immunostimulatory CpG DNA could induce potent anti‐tumor effects.^180^ Similarly, engineered myeloma cell‐derived exosomes expressing endogenous P1A tumor antigen and HSP70 are capable of stimulating dendritic cell maturation and T‐cell immune responses^181^ (Figure 6a).

Additionally, using immune cell‐derived exosomes to enhance anti‐tumor immunity is another research hotspot. Multiple studies showed that exosomes from dendritic cells loaded with tumor antigen are able to activate the cytotoxicity of tumor antigen‐specific CD8^+^ T cells and enhance anti‐tumor responses in animal models and human clinical trials.^101^, ^148^, ^182^, ^183^ Exosome from M1, but not M2 macrophages, enhances activity of lipid calcium phosphate (LCP) nanoparticle‐encapsulated Trp2 vaccine thus causes a stronger antigen‐specific cytotoxic T cell response, suggesting they could be used as a vaccine adjuvant.^184^ Exosomes from NK cells exert cytotoxic activity against different human tumor cells.^146^ The extracellular vesicles from activated CD8^+^ cells also prevent tumor progression by depleting the mesenchymal tumor stromal cells^185^ (Figure 6a). Not surprisingly, exosomes from the above‐mentioned immune cells should be employed in cancer immunotherapy in the near future.

Currently, liquid biopsy has emerged as a noninvasive and convenient approach for cancer diagnosis and prognostic monitoring. As mentioned above, due to the high stability and sufficient concentration in the circulation, exosomes have advantage in liquid biopsy compared with other sources such as circulating tumor cells (CTCs) and circulating tumor DNA (ctDNA).^186^ In addition, exosomes contain a variety of contents, such as protein and miRNAs, which can be implicated as biomarkers for diseases.^187^, ^188^ For example, GPC1^+^ circulating exosomes may serve as a potential non‐invasive diagnostic biomarker to detect early stages of pancreas cancer.^189^ Overwhelming studies have pointed out that exosomes contain high levels of miRNAs that contribute to immunoregulation, chemoresistance, and cancer metastasis in multiple tumor types.^99^, ^190^, ^191^ Numerous studies show that the exosomal RNAs can be used as diagnostic biomarkers as well. The miRNA signatures of TEXs show considerable promise as potential circulating diagnostic biomarkers in many types of cancer such as glioblastoma ovarian cancer and prostate cancer,^188^, ^192^, ^193^ as well as inflammatory liver diseases.^194^, ^195^ In addition to miRNAs, circular RNAs (circRNAs) were more abundant in exosomes derived from cancer cells and patients serum, which may serve as a new class of exosome‐based cancer biomarkers.^196^, ^197^

Interestingly, we noticed that exosomes isolated from pregnant women plasma and serum contain both maternal and fetal origin DNA,^198^ which proposes a possible application of exosomes for clinical utility in prenatal screening and diagnosis (Figure 6b).

TEXs prefer to promote tumor progression, thus blocking the biogenesis and release of TEXs seems to be a potential anti‐tumor strategy. GW4869, an inhibitor of nSMase2, has been discovered to block the ceramide synthesis.^70^ Treating tumor bearing mice with GW4869 reduces the lung metastasis and its combination with cisplatin and gefitinib increased the anti‐tumor effects.^199^ As the most frequently used genetic targets to downregulate exosomes production, Rab27a knockdown could inhibit exosomes secretion thus lead to a reduction of tumor growth.^200^, ^201^ TSG101, a protein involved on endosomes trafficking, was also thought to be a potential therapeutic target to interfere with exosome‐mediated communication in cancer.^202^ Furthermore, Cytochalasin D, an inhibitor of different endocytosis, can inhibit exosomes uptake, which also downregulate the exosomes biogenesis.^203^ In summary, antagonizing the synthesis, release and uptake of tumor exosomes benefits cancer therapy (Figure 6c).

## 10 Conclusions and Future Perspectives

### 10.1 The Mechanisms Underlying the MVBs' Sorting Remains Mysterious

There is no doubt that functions of exosomes are determined by their specific content, in other words, depending on the cargos that are specifically delivered. Although many proteins have been identified in exosomes, little is known about how they were chosen and sorted into the exosomes, what particular post‐translational modification (PTM) is required for or contributes to exosomal accumulation of proteins. Practically, some but not all of the internalized membrane proteins are frequently found to be phagocytosed into endosome and finally secreted into exosomes, which is likely to be the result of a multi‐step protein trafficking/sorting that continuously occurred during transport of intracellular vesicles. The MVB sorting process plays a critical role in facilitating the degradation of membrane proteins within the hydrolytic lumen of the lysosome/vacuole. Although in the past 10 years, the basic framework by which MVB sorting to lysosome has been elucidated,^204^ but how MVB sorting could be switched for producing exosomes remain mysterious. The mechanisms that sort MVB to the plasma membrane and the lysosome are largely unclear, but the existence of a decision point between those two fates suggests that inhibition of one pathway will increase the other.^205^, ^206^ Cells might compensate for lysosome malfunction by disposal of potentially toxic cargos into exosomes, thus future studies of the molecular mechanisms underlying the MVBs' trafficking may advance current understanding on how pathogenic proteins, lipids or infectious agents accumulate outside of cells.^206^

### 10.2 Exploration of DNA in EVs

Exosomes contain a small amount of DNA, including single‐stranded DNA, double‐stranded DNA, genomic DNA, mitochondrial DNA, and even reverse‐transcribed complementary DNAs.^45^, ^46^, ^207^ Whether the source of DNA in exosome is nucleus, mitochondria, or cytoplasm is still unknown. Unlike other exosomal cargoes, it is not clear whether selective packaging of specific DNA into exosomes exists. What is the function of exosomal DNAs also needs further explanation. For example, TEXs produced by irradiated mouse breast cancer cells (RT‐TEX) transfer dsDNA to DCs and stimulate STING‐dependent activation of type I IFN (IFN‐I), resulting in eliciting tumor‐specific CD8 T‐cell responses.^208^ Similarly, recent study has shown that T cell‐derived exosomes contain genomic and mitochondrial DNA (mtDNA) which is transmitted from the T cell to the DC to induce antiviral responses.^209^ Transfer of exosomal DNA activates the cGAS/STING cytosolic DNA‐sensing pathway and increases the expression of IRF3‐dependent interferon regulated genes in DCs,^209^ and transfer of mtDNA acts as an oncogenic signal promoting an exit from dormancy of therapy‐induced cancer stem‐like cells.^210^ Moreover, exosome derived from senescent cells contain chromosomal DNA fragments discarded as cellular garbage bags from cells.^211^ These results indicated that exosome secretion might play crucial roles in maintaining cellular homeostasis by removing harmful cytoplasmic DNA from cells, which prevents ATM/ATR‐dependent DNA damage response and aberrant innate immune responses.

Although there are many studies on exosomal DNA, the heterogeneity of extracellular vesicles and nanoparticles, as well as differences in purification strategies make the analysis of exosomes confusing. As mentioned above, a recent study breaks the general view that exosomes are the carriers of extracellular DNA secretion.^6^

This work suggests that double‐stranded DNA (dsDNA) and DNA‐bound histones do not exist in exosomes or any other type of sEV. In view of the increasing interest in extracellular DNA as a marker of disease in liquid biopsy, it is necessary to reassess the actual measurement results. However, compared with traditional exosome isolation methods, the revised exosome isolation with greater precision used in this study is more costly and less efficient. Therefore, there is a great need for more standardized isolation and purification techniques of exosomes, or even a revision of the current classification and nomenclature.^212^ In summary, the heterogeneity of EVs and the presence of non‐vesicular extracellular nanoparticles pose major obstacles to our understanding of the composition and functional properties of different secretory components. A more accurate understanding of the correct extracellular components of RNA, DNA, and proteins and their secretion mechanisms are essential for identifying biomarkers and designing future drug interventions.

### 10.3 The Challenges in Exosome‐Based Therapy

Although exosomes have made great achievements in applications, challenges still remain. Since exosomes can be utilized as clinical biomarkers, vaccines, or drug delivery devices, more accurate and standardized purification method is urgently needed. Besides, for an achievement of better immunotherapy or vaccination based on exosomes, the antigen‐loading efficiency of exosomes must be improved. Another challenge is to generate large‐scale production of exosomes for clinical application. Although a process for production of good manufacturing practice (GMP)‐grade exosomes derived from mesenchymal stem/stromal cells has been reported, this technique still requires expansion to other different cell types.^213^ Moreover, what are the most suitable cells for producing clinical‐grade exosomes remains to be further investigated. In addition, superlative exosome‐based therapy could combine with other anti‐tumor treatments, which will be broad potential. Through the study of exosomes, more widespread therapeutic applications can be proposed.

## Conflict of Interest

The authors declare no conflict of interest.
